# Murine Gut Microbiome Meta-analysis Reveals Alterations in Carbohydrate Metabolism in Response to Aging

**DOI:** 10.1128/msystems.01248-21

**Published:** 2022-04-11

**Authors:** Xiaomeng You, Ushashi C. Dadwal, Marc E. Lenburg, Melissa A. Kacena, Julia F. Charles

**Affiliations:** a Department of Orthopaedic Surgery, Brigham and Women’s Hospital, Harvard Medical School, Boston, Massachusetts, USA; b Orthopaedic Surgery, Indiana University School of Medicine, Indianapolis, Indiana, USA; c Department of Medicine, Boston University School of Medicine, Boston, Massachusetts, USA; d Department of Medicine, Brigham and Women’s Hospital, Harvard Medical School, Boston, Massachusetts, USA; Duke University School of Medicine

**Keywords:** aging, murine gut microbiome, meta-analysis, carbon metabolism

## Abstract

Compositional and functional alterations to the gut microbiota during aging are hypothesized to potentially impact our health. Thus, determining aging-specific gut microbiome alterations is critical for developing microbiome-based strategies to improve health and promote longevity in the elderly. In this study, we performed a meta-analysis of publicly available 16S rRNA gene sequencing data from studies investigating the effect of aging on the gut microbiome in mice. Aging reproducibly increased gut microbial alpha diversity and shifted the microbial community structure in mice. We applied the bioinformatic tool PICRUSt2 to predict microbial metagenome function and established a random forest classifier to differentiate between microbial communities from young and old hosts and to identify aging-specific metabolic features. In independent validation data sets, this classifier achieved an area under the receiver operating characteristic curve (AUC) of 0.75 to 0.97 in differentiating microbiomes from young and old hosts. We found that 50% of the most important predicted aging-specific metabolic features were involved in carbohydrate metabolism. Furthermore, fecal short-chain fatty acid (SCFA) concentrations were significantly decreased in old mice, and the expression of the SCFA receptor *Gpr41* in the colon was significantly correlated with the relative abundances of gut microbes and microbial carbohydrate metabolic pathways. In conclusion, this study identified aging-specific alterations in the composition and function of the gut microbiome and revealed a potential relationship between aging, microbial carbohydrate metabolism, fecal SCFA, and colonic *Gpr41* expression.

**IMPORTANCE** Aging-associated microbial alteration is hypothesized to play an important role in host health and longevity. However, investigations regarding specific gut microbes or microbial functional alterations associated with aging have had inconsistent results. We performed a meta-analysis across 5 independent studies to investigate the effect of aging on the gut microbiome in mice. Our analysis revealed that aging increased gut microbial alpha diversity and shifted the microbial community structure. To determine if we could reliably differentiate the gut microbiomes from young and old hosts, we established a random forest classifier based on predicted metagenome function and validated its performance against independent data sets. Alterations in microbial carbohydrate metabolism and decreased fecal short-chain fatty acid (SCFA) concentrations were key features of aging and correlated with host colonic expression of the SCFA receptor *Gpr41*. This study advances our understanding of the impact of aging on the gut microbiome and proposes a hypothesis that alterations in gut microbiota-derived SCFA-host GPR41 signaling are a feature of aging.

## INTRODUCTION

The human gut harbors a complex ecosystem of microorganisms, collectively referred to as the gut microbiota. The initial colonization of the gut microbiota is generally thought to be established at birth, developing toward a stable adult-like microbial community structure by the age of 3 years (1). The gut microbiota develops with human hosts and has a variety of biological functions in human health and disease. Increasing evidence suggests that the gut microbiota plays important roles in the development and maturation of the immune system, food digestion and nutrient metabolism, gut endocrine regulation, neurological signaling, defense against pathogens, and elimination of toxins (2).

Aging is a time-dependent multifactorial process characterized by functional declines in a variety of biological and physiological systems and an increased risk for major human chronic diseases (3). During the aging process, remarkable aging-related gut microbial alterations have been observed in human studies across various populations (4, 5). However, gut microbiome profiles across different elderly populations vary widely. A number of studies have found that the aged human gut microbiome is characterized by increased proportions of opportunistic pathogens (e.g., *Clostridioides*) and decreases in health-promoting bacteria (e.g., bifidobacteria and lactobacilli) (4–8). Moreover, several studies conducted on extreme-aged people (centenarians) show that the microbial profiles of extreme-aged people differ from those of old adults, potentially identifying a healthy aging microbial pattern (9–11). This healthy aging microbiome is characterized by the depletion of core microbial taxa (e.g., *Bacteroides* and *Prevotella* spp.) and becomes increasingly unique to individuals (9). The heterogeneity of findings in human aging studies indicates that multiple gut microbiome patterns of aging exist.

As we age, our lifestyle and diet are likely to change due to a reduction in our physiological systems. Aging is often accompanied by reduced food diversity, especially in fiber-containing foods (e.g., fruits and vegetables), as well as an increased risk of malnutrition. A healthy and diverse diet is positively related to a more diverse gut microbiota in old people (12). Dietary supplementation of prebiotics (e.g., inulin) leads to a higher abundance of beneficial bacterial groups (e.g., *Bifidobacterium*) in old people (13). On the other hand, malnutrition is associated with an increased abundance of *Clostridiales* taxa, which has been associated with increased frailty in aged individuals (12, 14). Thus, diet may account for at least a portion of the variation in microbial composition among different elderly populations. Moreover, aged populations are more likely to experience hospitalization, infections, and exposure to antibiotics and other medications (15), which would also significantly impact the gut microbiome composition. The gut microbiota profile of old people in long-term-care facilities differs from those of community-dwelling subjects, with a decrease in microbial diversity and a loss of community-associated taxa (e.g., *Coprococcus* and *Roseburia*) (12). Thus, the interpretation of studies correlating age with changes in microbial communities is complex due to the various factors that would potentially impact and confound our understanding of aging-associated gut microbiome alterations. Systemically investigating and separating the effects of aging and exposures on gut microbiota composition and function are difficult in humans but may be accomplished in experimental animal models.

Research animals, such as mice (Mus musculus), are maintained under standardized conditions, with uniform diet, bedding, dark-light cycles, and other environmental exposures. Thus, experimental mice are a useful model system to study aging-specific changes in the host-microbiome interaction. Aging-related gut microbial alterations have been investigated in mice in several studies (16–20). A distinct gut microbial composition and a structure shift between young and old mice have been found, which partially resemble the changes observed in human studies. However, when we examined changes in specific taxa or functional alterations, inconsistent or conflicting results were common. For example, the *Firmicutes*-to-*Bacteroidetes* ratio (F/B ratio), an index often used to describe gut microbiome profiles, was increased in aged mice in a study by Kim et al. (21), while it was decreased in a study by Zhang et al. (22) and unchanged in a study by Langille et al. (23).

Inconsistent findings from different mouse studies make it difficult to come to a consensus regarding aging-specific microbial alterations, a prerequisite to identifying microbiome-based therapeutic targets for aging-related diseases. In this study, we performed a meta-analysis of publicly available 16S rRNA gene sequencing data of studies investigating the effect of aging on the gut microbiome in mice. By integrating aging-related microbiome alterations across 5 independent studies, we hoped to identify age-specific changes in community composition or function common to all studies and then validate these in additional data sets. In brief, we reanalyzed raw sequencing data from all studies using the same pipeline. We then applied the bioinformatic tool PICRUSt2 (Phylogenetic Investigation of Communities by Reconstruction of Unobserved States) to predict microbial metagenome function and established a random forest classifier to differentiate young and old age and identify aging-specific metabolic features. We then validated our random forest classifier against one 16S rRNA gene sequencing data set and one whole-genome metagenomic sequencing data set from young and old mice generated by our laboratory. Our analysis identified an increase in gut microbial diversity with age and revealed aging-associated alterations in microbial carbohydrate metabolism that significantly correlated with colonic *Gpr41* expression, revealing how aging affects one mechanism of host-microbe interactions, that is, gut microbiome-produced short-chain fatty acids (SCFA) acting through their receptors.

## RESULTS

### Microbial alpha diversity increased with aging.

**FIG 1:**
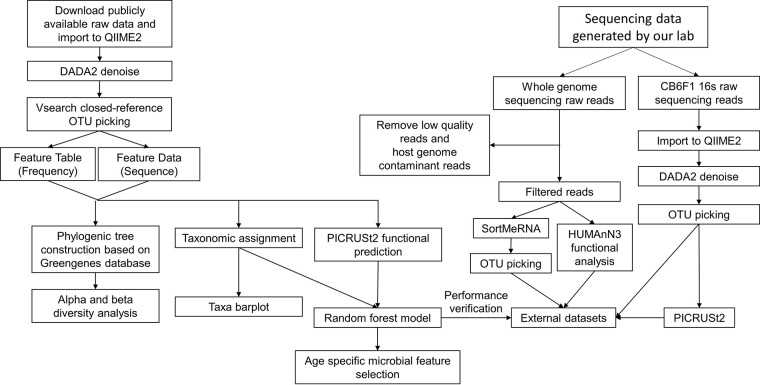
Bioinformatic analysis pipeline. The raw 16S rRNA gene sequencing data were downloaded and imported into QIIME2 for data processing. DADA2 was used to denoise reads. OTU picking was performed by the Vsearch closed-reference method. The resulting feature table and feature data were then used for phylogenic tree construction and taxonomy annotation, followed by diversity analysis and taxon bar plot visualization. Functional prediction was performed by PICRUSt2. A random forest classifier was established based on predicted functional pathways to differentiate young- and old-age gut microbiomes and identify aging-specific metabolic features. The random forest classifier was then verified by two external data sets generated by us. The CB6F1 16S rRNA gene sequencing data were analyzed according to the same analysis pipeline. The whole-genome sequencing data were first filtered to remove low-quality and host genome contaminant reads, followed by SortMeRNA and OTU picking for taxonomy characterization and HUMAnN3 for functional analysis.

**FIG 2:**
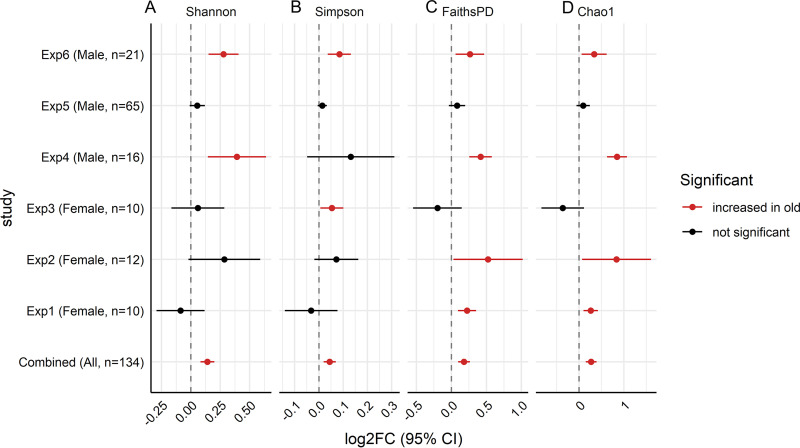
Forest plots of alpha diversity metrics. Shannon (A), Simpson (B), Faith’s PD (C), and Chao1 (D) indices demonstrated an increase in alpha diversity in response to aging. Analysis of the combined data sets was performed using a linear mixed-effects model using the formula log_2_FC ∼ age + (1|study). A *P* value of <0.05 was considered statistically significant.

The bioinformatic analysis pipeline used to process and analyze the raw sequencing data is shown in Fig. 1 and described in detail in Materials and Methods. Common metrics for alpha diversity, including Shannon, Simpson, Faith’s phylogenetic diversity (PD), and Chao1 indices, were calculated. Due to the heterogeneity of studies, including varied sample processing, sequencing platforms, variable regions, study sizes, and sequencing depths across data sets (detailed in Table 1 and Fig. S2 in the supplemental material), the data were visualized by log_2_ fold changes (log_2_FCs) of old to young with 95% confidence intervals (CIs) on a per-data-set basis (Fig. 2). With the exception of experiment 5 (Exp5), each data set had one or more alpha diversity indices that were significantly increased in the aged mice (Fig. 2). When considering all data sets together, all 4 alpha diversity indices were significantly increased in the aged mice (Fig. 2) (Shannon 95% CI, 0.082, 0.200; Simpson 95% CI, 0.020, 0.070; Chao1 95% CI, 0.147, 0.389; Faith’s PD 95% CI, 0.097, 0.265 [linear mixed-effect model adjusted for study, *P* < 0.001 for each diversity index]).

**TABLE 1:** Summary of study characteristics

Data set	Sex	Age (mo)		Sequencing region	No. of samples		Database/accession no.	Reference
		Young	Old		Young	Old		
Exp1	Female	2	26	V4	5	5	SRA/PRJNA311095	M. N. Conley et al. (16)
Exp2	Female	1.5–2	18.5	V4	6^*a*^	6^*a*^	SRA/PRJNA588787	H. C. Barreto et al. (17)
Exp3	Female	3	22	V3-V4	5^*b*^	5^*b*^	ENA/PRJEB31652	M. Stebegg et al. (18)
Exp4	Male	4	24	V3-V4	8	8^*c*^	SRA/PRJNA450595	B. van der Lugt et al. (20)
Exp5	Male	5–6	18–20	V4-V5	38^*d*^	27^*d*^	SRA/PRJNA400638	J. D. Hoffman et al. (19)
Exp6	Male	3	22	V3-V4	7^*b*^	14^*b*^	ENA/PRJEB31652	M. Stebegg et al. (18)
CB6F1	Male	3	24	V4	8	8	SRA/PRJNA737742	TBD^*e*^
Metagenomics	Male	3	26	Whole genome	10	10	SRA/PRJNA739153	This study

a Only control young and old data were included.

b Only control young and old C57BL/6 data were included.

c Only fresh fecal samples were included.

d Low-sequencing-depth data were excluded.

e TBD, to be determined (You X, Yan J, Herzog J, Campbell R, Hoke A, Hammamieh R, Sartor RB, Kacena MA, Chakraborty N, Charles JF, manuscript in preparation).

10.1128/mSystems.01248-21.2 FIG S2 Sequencing reads across samples (A) and studies (B). Samples were rarefied to 10,000 reads as indicated by the red dotted line in panel B. Download FIG S2, TIF file, 2.1 MB. Copyright © 2022 You et al. 2022 You et al. https://creativecommons.org/licenses/by/4.0/ This content is distributed under the terms of the Creative Commons Attribution 4.0 International license.

**FIG 3:**
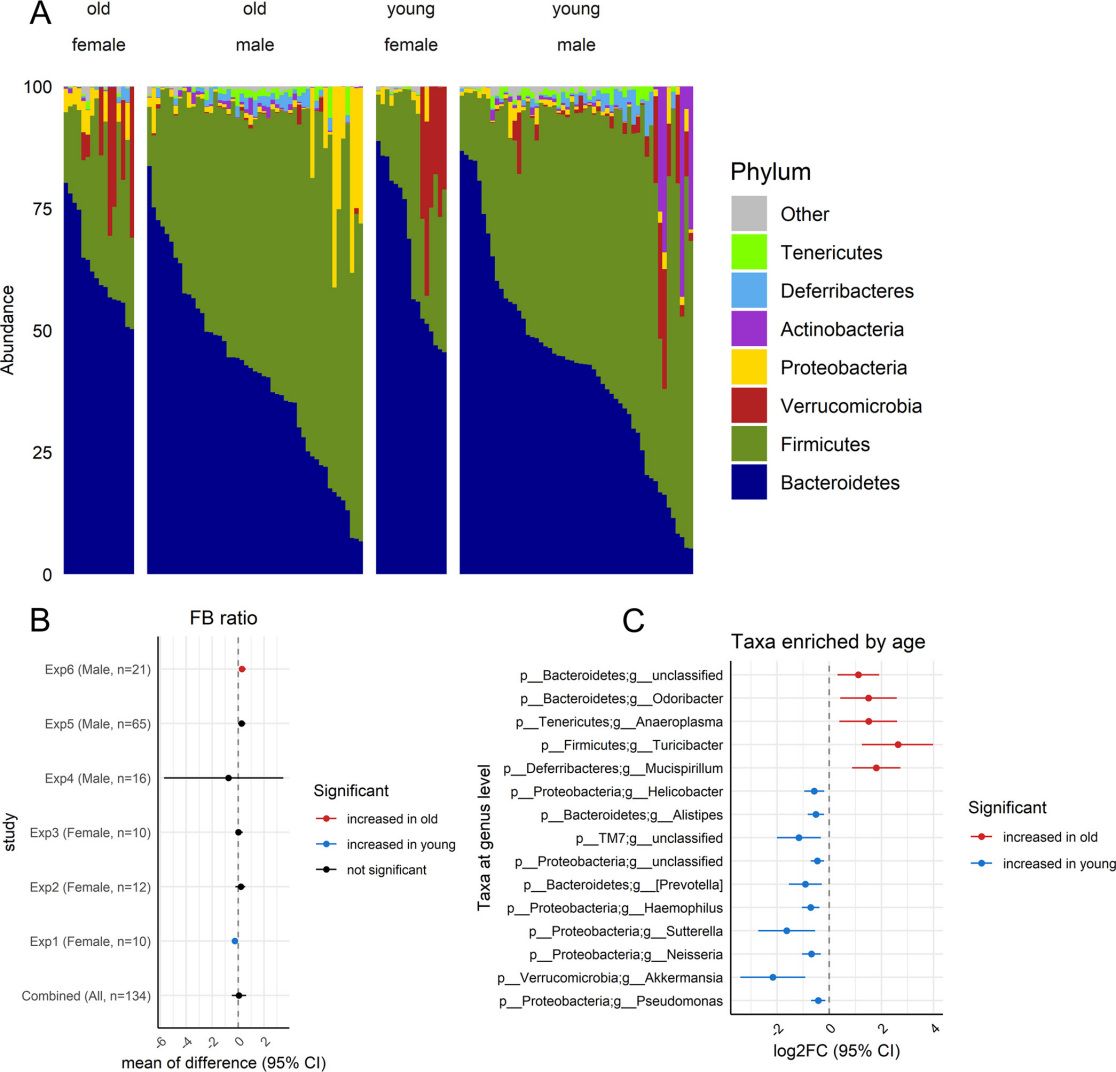
Comparative compositions of the gut microbiomes of young and old mice. (A) Relative abundances of taxa at the phylum level show that *Firmicutes* and *Bacteroidetes* dominate the microbial communities in both young and aged mice. (B) A forest plot of the ratio of the *Firmicutes* phylum to the *Bacteroidetes* showed no significant alteration between young and old mice. (C) Taxa significantly enriched by age on combined data sets. For panels B and C, the combined analysis was performed using a linear mixed-effects model using the formula log_2_FC ∼ age + (1|study). The Benjamini-Hochberg method was used to correct for multiple comparisons. A *P* value of <0.05 was considered statistically significant.

The human gut microbiota is mostly composed of two dominant bacterial phyla, *Firmicutes* and *Bacteroidetes* (24). Contradictory results regarding the direction of F/B ratio alterations in response to aging have been reported (21–23). Thus, we calculated the relative abundance of taxa at the phylum level and the F/B ratio. As expected, *Firmicutes* and *Bacteroidetes* dominated the microbial community in both young and aged mice (Fig. 3A). However, no alteration of the F/B ratio was found in the aged mice compared to the young mice when considering all the data in aggregate (Fig. 3B) (95% CI, −0.49, 0.64 [linear mixed-effect model adjusted for study, *P* > 0.05]) or in 4 of the 6 data sets. Consistently, we found that the relative abundances of these two phyla were not altered with aging (Table S3). Instead, the subdominant taxon *Deferribacteres* was significantly enriched and the subdominant taxon *Verrucomicrobia* was significantly decreased by old age (Table S3). At the genus level, we found a total of 15 taxa that were significantly enriched by young or old age, including *Mucispirillum* (log_2_FC 95% CI, 0.88, 2.73 [Benjamini-Hochberg {BH}-corrected *P* < 0.05) and *Akkermansia* (log_2_FC 95% CI, −3.41, −0.92 [BH-corrected *P* < 0.05]), which are the subgroups under the phyla *Deferribacteres* and *Verrucomicrobia* (Fig. 3C).

10.1128/mSystems.01248-21.9 TABLE S3 Results of the enrichment analysis on taxa at the phylum level and genus level. Download Table S3, XLSX file, 0.02 MB. Copyright © 2022 You et al. 2022 You et al. https://creativecommons.org/licenses/by/4.0/ This content is distributed under the terms of the Creative Commons Attribution 4.0 International license.

### Aging shifts gut microbiome structure.

**FIG 4:**
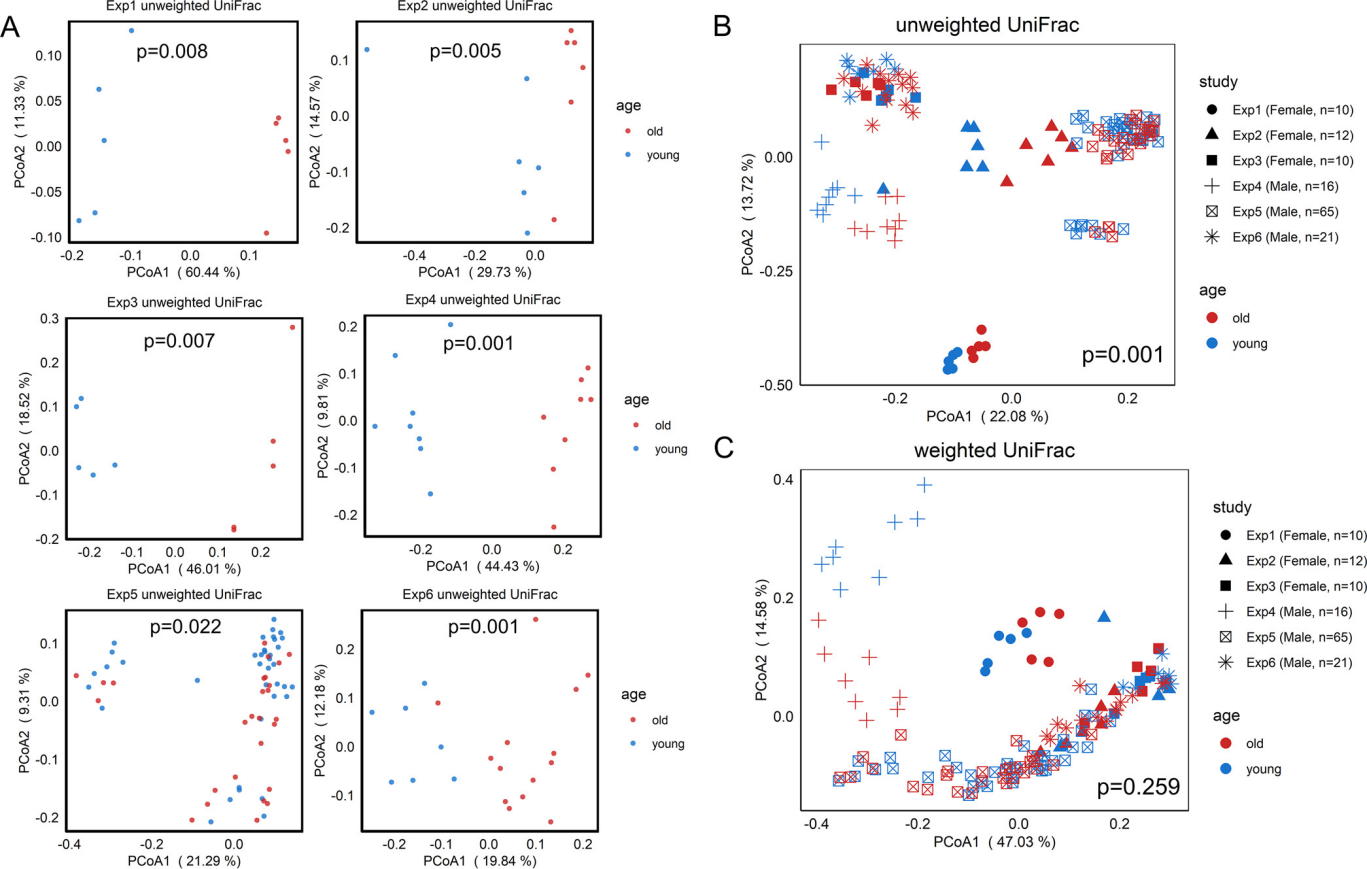
Age-related shifts in microbial community structure. The effect of age on community structure for each study individually and for the combined studies was assessed by principal-coordinate analysis (PCoA) of the beta diversity measures unweighted and weighted UniFrac distances. Significance was determined by PERMANOVA with 999 permutations. (A and B) PCoA plots for unweighted UniFrac distances for each study (A) and the aggregate data (B) show significant shifts in the gut microbiome between the young and old mice. (C) PCoA of weighted UniFrac distances for aggregated data did not reveal a significant difference.

The beta diversity indices unweighted and weighted UniFrac distances were calculated and visualized with principal-coordinate analysis (PCoA) plots per data set and for all data sets in aggregation (Fig. 4 and Fig. S3). Permutational multivariate analysis of variance (PERMANOVA) with 999 permutations was used to test for significant differences between young and old mice. Based on unweighted UniFrac distance matrices, all data sets demonstrated a significant shift in the gut microbiome between the young and old mice (*P* < 0.05) (Fig. 4A). When all the data sets were aggregated, a significant age-dependent alteration of the microbial community structure was observed based on unweighted UniFrac distance matrices (*P* = 0.001) (Fig. 4B). However, based on weighted UniFrac distance matrices, no significant difference was observed in 2 of 6 data sets (Fig. S3) or in the aggregate data (*P* = 0.259) (Fig. 4C). These results indicate that rare taxa rather than dominant taxa are primarily responsible for the microbial structure differences between young and old mice.

10.1128/mSystems.01248-21.3 FIG S3 Principal-coordinate analysis (PCoA) based on weighted UniFrac distances for each study. Download FIG S3, TIF file, 2.9 MB. Copyright © 2022 You et al. 2022 You et al. https://creativecommons.org/licenses/by/4.0/ This content is distributed under the terms of the Creative Commons Attribution 4.0 International license.

### Altered gut microbial carbohydrate metabolism in response to aging.

Random forest was used to differentiate young and old age based on operational taxonomic units (OTUs) and predicted metagenomic function by PICRUSt2 to identify aging-specific microbial signatures. An iterative random forest (iRF) algorithm was performed to identify features that were stable across 10 iterations, of which the iteration with the smallest out-of-bag (OOB) error rate was chosen as the final classifier for further analysis.

**FIG 5:**
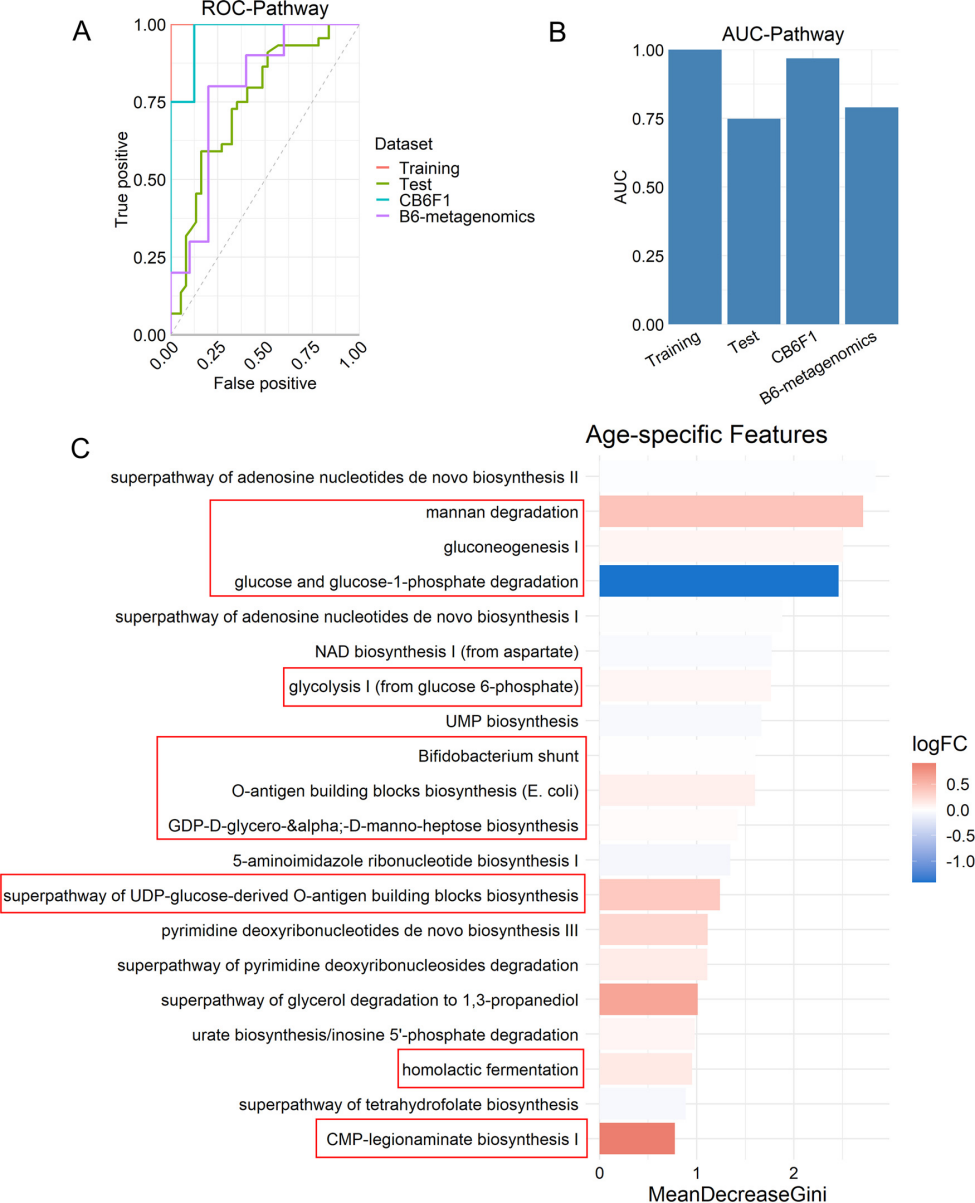
Random forest classifier differentiates microbiomes of young and old mice. (A) Receiver operating characteristic (ROC) curve for the random forest classifier showing performance against the test data set (green) and external data sets, CB6F1 16S (blue) and B6 metagenomic (purple). (B) Area under the ROC curve (AUC). (C) The top 20 aging-specific features of the classifier based on ranked mean decreases in Gini impurities. The color bar shows the log_2_ fold changes (logFC) of old versus young. Features boxed in red are involved in carbohydrate metabolism.

We calculated the area under the receiver operating characteristic (ROC) curve (AUC) to estimate the performance of the random forest classifier. The AUC values of the model based on predicted metagenomic function for the test data set, the external CB6F1 16S data set, and the external B6 metagenomics data set were 0.75, 0.97, and 0.79, respectively (Fig. 5A and B). Interestingly, the model based on OTUs did not perform as well as the one based on predicted metagenomic function, which achieved AUC values of between 0.46 and 0.73 (Fig. S5). This result could be explained by microbial functional redundancy (25). That is, despite large variations in taxonomic composition across studies of mice from different vivariums, consistent changes in functional capacity in response to aging can be observed. Thus, metagenomic functional features selected by the random forest model might be more reliable than taxonomic features to stratify young and old mice and exhibit similar alterations in response to aging across studies.

10.1128/mSystems.01248-21.5 FIG S5 Performance of the random forest model on OTUs. (A) Receiver operating characteristic (ROC) curve for the random forest classifier showing performance against the test data set (green) and external data sets, CB6F1 16S (blue) and B6 metagenomic (purple). (B) Area under the receiver operating characteristic curve (AUC). Download FIG S5, TIF file, 0.7 MB. Copyright © 2022 You et al. 2022 You et al. https://creativecommons.org/licenses/by/4.0/ This content is distributed under the terms of the Creative Commons Attribution 4.0 International license.

Next, aging-specific metagenomic features were selected based on the ranked mean decrease in Gini impurities (mean decrease Gini) (Fig. 5C), which measures the importance of a feature for classification in the random forest model. Interestingly, we found that 50% of the 20 most important features are involved in carbohydrate metabolism, including mannan degradation, gluconeogenesis I, and glucose and glucose-1-phosphate degradation. We also performed an enrichment analysis on predicted metagenomic function pathways across the studies (linear mixed-effect model adjusted for study). Despite the different methods performed, we found that 13 out of 25 significantly enriched pathways were involved in carbohydrate metabolism (Fig. S6) (BH-corrected *P* < 0.05). Taken together, alterations in the carbohydrate metabolism potential of the gut microbial metagenome are a key distinguishing feature of aging.

10.1128/mSystems.01248-21.6 FIG S6 Predicted metagenomic functional pathways significantly enriched by age on combined data sets. The analysis was performed by a linear mixed-effects model using the formula log_2_FC ∼ age + (1|study). Features boxed in red are involved in carbohydrate metabolism. The Benjamini-Hochberg method was used to correct for multiple comparisons. A *P* value of <0.05 was considered statistically significant. Download FIG S6, TIF file, 1.3 MB. Copyright © 2022 You et al. 2022 You et al. https://creativecommons.org/licenses/by/4.0/ This content is distributed under the terms of the Creative Commons Attribution 4.0 International license.

### Decreased fecal SCFA in response to aging.

**FIG 6:**
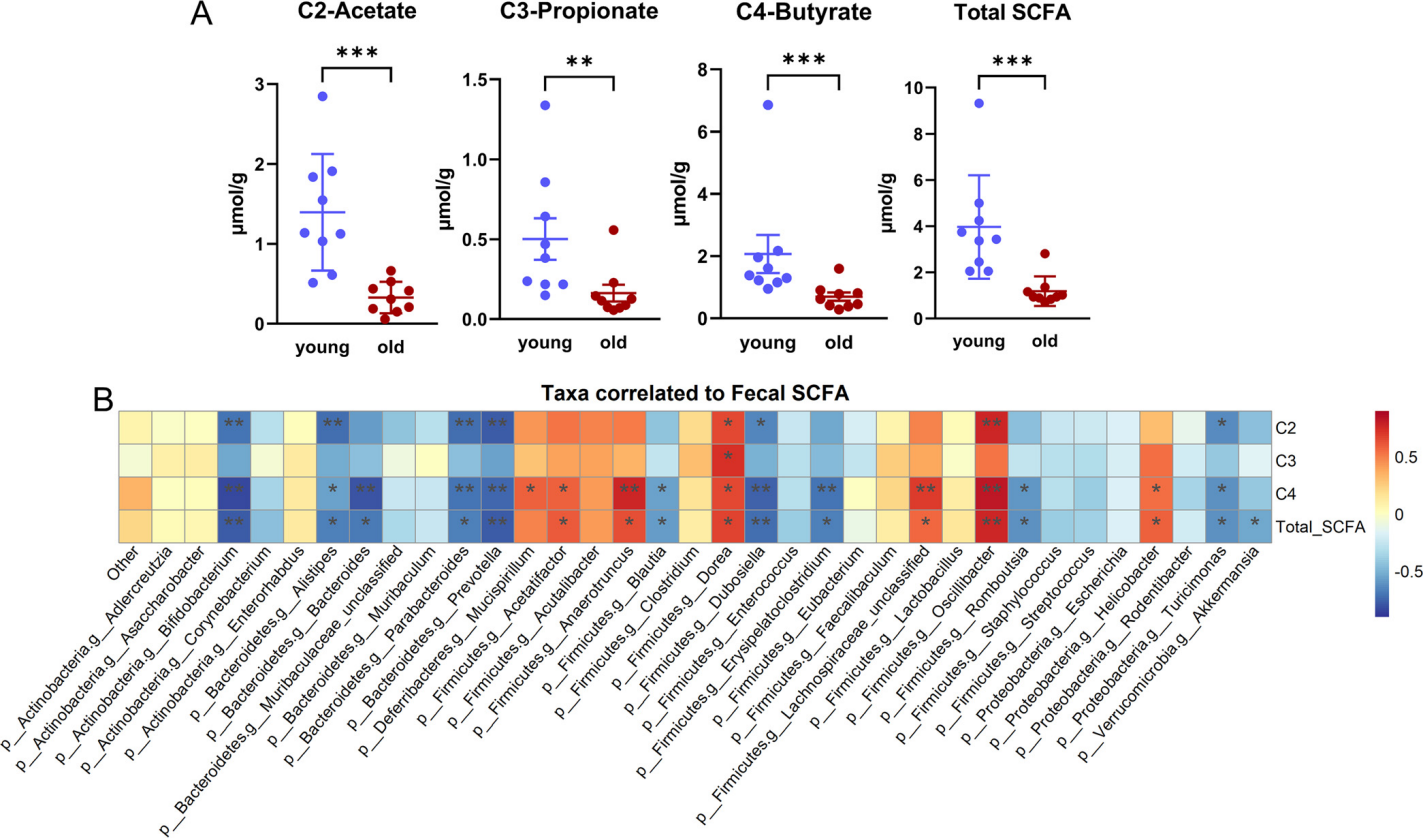
Fecal SCFA decrease with aging. (A) Fecal acetate, propionate, butyrate, and total SCFA in young and old mice. (B) Taxa at the genus level correlated with fecal SCFA concentrations. The Benjamini-Hochberg method was used to correct for multiple comparisons. *, *P* < 0.05; **, *P* < 0.01; ***, *P* < 0.001.

Given the altered microbial carbohydrate metabolism metagenome with aging described above, we investigated whether fecal short-chain fatty acids (SCFA), the major end products of microbial carbohydrate fermentation, were changed with aging as well. We measured three primary SCFA concentrations in fecal samples from young and old mice used to generate the metagenomic data set. Fecal acetate (*P* < 0.001), propionate (*P* < 0.01), butyrate (*P* < 0.001), and total SCFA (*P* < 0.001) concentrations were significantly decreased with age (Fig. 6A). The decreased SCFA concentrations with aging in our cohort are consistent with previous studies in humans, mice, and rats (26–29).

Additionally, Spearman correlation analysis was performed between fecal SCFA and taxa at the genus level (Fig. 6B). We found several taxa considered to be SCFA producers or beneficial microbes (30–33) that were positively correlated with one or more fecal SCFA, including *Dorea* (BH-corrected *P* < 0.05 for acetate, propionate, butyrate, and total SCFA), *Oscillibacter* (BH-corrected *P* < 0.05 for acetate, butyrate, and total SCFA), *Mucispirillum* (BH-corrected *P* < 0.05 for butyrate), *Acetatifactor* (BH-corrected *P* < 0.05 for butyrate and total SCFA), and *Anaerotruncus* (BH-corrected *P* < 0.05 for butyrate and total SCFA). Additionally, several taxa were significantly anticorrelated with one or more fecal SCFA, including *Bifidobacterium*, *Alistipes*, *Parabacteroides*, *Prevotella*, *Dubosiella*, *Turicimonas*, *Bacteroides*, *Blautia*, *Erysipelatoclostridium*, and *Romboutsia* (BH-corrected *P* < 0.05 for one or more metabolites). The negative correlation between *Bifidobacterium* and fecal SCFA is unexpected as *Bifidobacterium* is well known as a beneficial microbe that metabolizes dietary fibers to acetate and lactate via the bifid shunt (34). *Bifidobacterium* may alter metabolic pathways in response to environmental conditions (e.g., the availability of carbohydrates) (35). For example, *Bifidobacterium* could shift toward formate production at the expense of lactate when there is an increased need for ATP (35, 36). Furthermore, a correlation does not necessarily indicate a direct relationship between a given microbe and SCFA generation. Further studies are needed to investigate the direct contribution of particular microbes to fecal SCFA via *in vitro* microbial community fermentation or investigations *in vivo* utilizing gnotobiotic mice.

### Correlation of SCFA receptor *Gpr41* gene expression with carbohydrate metabolism pathways.

**FIG 7:**
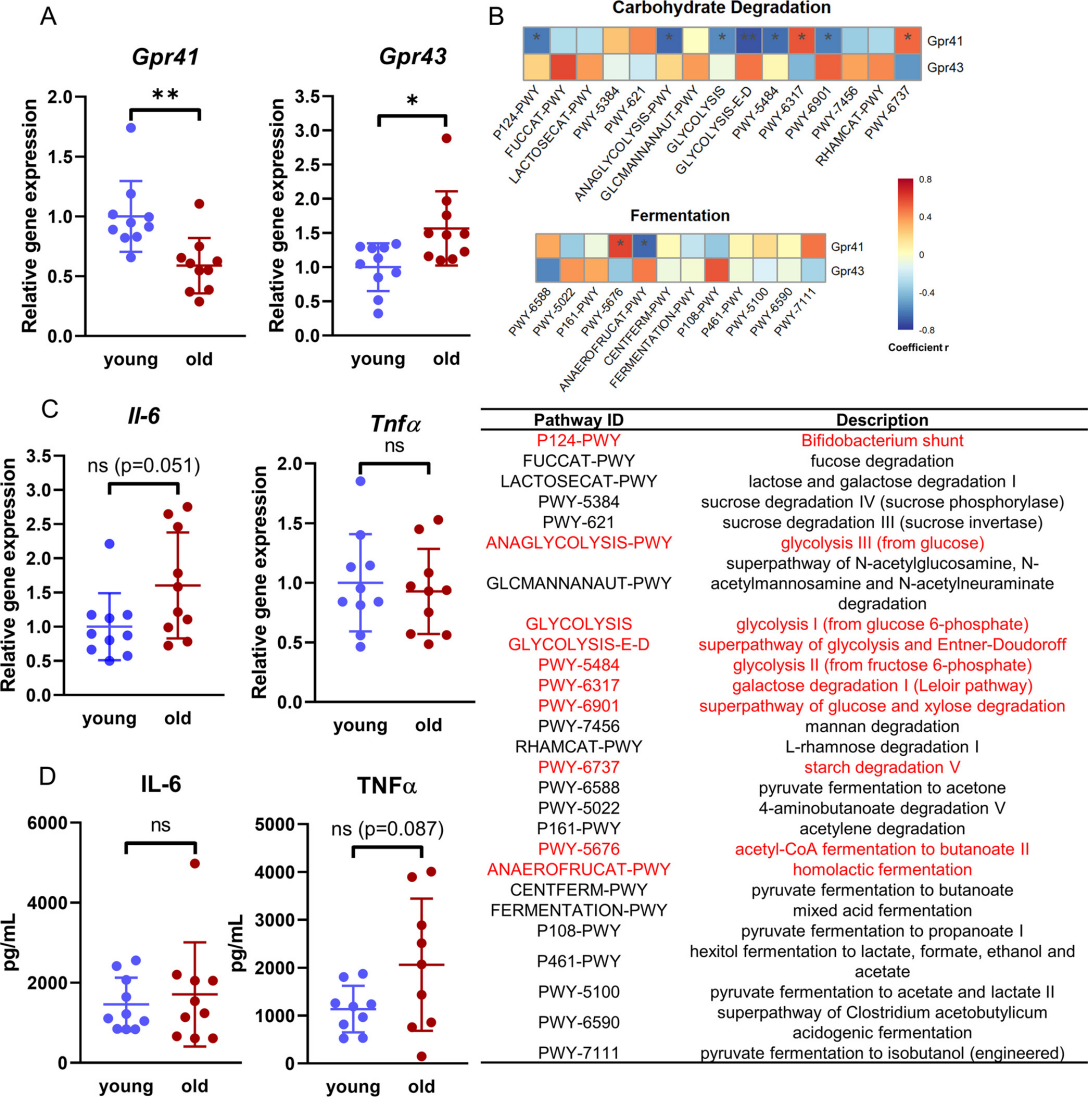
Correlation of *Gpr41* and microbial carbohydrate metabolism. (A) Relative expression of *Gpr41* and *Gpr43* in the colons of young versus old mice. (B) Correlation of colonic *Gpr41* expression with carbohydrate degradation and fermentation pathways. Significantly correlated pathways are highlighted in red text. (C and D) Comparison of IL-6 and TNF-α in young versus old mice. (C) Colonic *Il-6* and *Tnf*α expression; (D) serum IL-6 and TNF-α. ns, not significant; *, *P* < 0.05; **, *P* < 0.01.

G-protein-coupled receptor 41 (GPR41) and GPR43 are the primary receptors that sense SCFA and mediate the biological effect of SCFA on host health (37). To assess whether the decreased fecal SCFA with aging would impact the expression level of SCFA receptors, we measured *Gpr41* and *Gpr43* gene expression in the colons of young and aged mice. *Gpr41* expression was significantly decreased while *Gpr43* expression was significantly increased in the aged mice compared to young mice (*P* < 0.05) (Fig. 7A). *Gpr41* expression was significantly correlated with carbohydrate degradation and fermentation pathways, including the *Bifidobacterium* shunt, glycolysis, and homolactic fermentation (Spearman rank correlation, BH-corrected *P* value of <0.05) (Fig. 7B). Interestingly, these pathways were identified as aging-specific metabolic features by our random forest classifier. Additionally, *Gpr41* expression trended toward correlation with fecal acetate (BH-corrected *P* = 0.085) (Table S4) and propionate (BH-corrected *P* = 0.058) (Table S4) and was negatively correlated with two taxa at the genus level (*Alistipes* and *Parabacteroides*) (BH-corrected *P* < 0.05) (Table S4). Interestingly, these two taxa were also negatively correlated with fecal SCFA (BH-corrected *P* < 0.05) (Fig. 6B), indicating a cooccurrence of alterations in the gut microbial community, microbial SCFA, and host GPR41 with aging. Thus, we propose that the gut microbiome shifts as the host ages, resulting in altered microbial carbohydrate metabolism that could in turn impact colon SCFA concentrations, which could feed forward to impact *Gpr41*/*43* expression and host physiology.

10.1128/mSystems.01248-21.10 TABLE S4 Results of correlation analysis between *Gpr41*/*43* expression and fecal SCFA or taxa. Download Table S4, XLSX file, 0.01 MB. Copyright © 2022 You et al. 2022 You et al. https://creativecommons.org/licenses/by/4.0/ This content is distributed under the terms of the Creative Commons Attribution 4.0 International license.

As SCFA have been reported to influence multiple facets of inflammation, through their actions both as G-protein-coupled receptor ligands and as inhibitors of histone deacetylases (38), we measured colon gene expression and serum levels of interleukin-6 (IL-6) and tumor necrosis factor alpha (TNF-α) (Fig. 7C and D). A trend of increased *Il-6* colon gene expression (*P* = 0.051) and serum TNF-α levels (*P* = 0.087) was found in the aged mice, indicating increased inflammation in the aged mice, which is consistent with the general observation of low-grade chronic inflammation status in the elderly (inflammaging) (39).

## DISCUSSION

Cooperation between the gut microbiome and the host is finely tuned, with the gut microbiome constantly integrating signals from the host and responding throughout the lifetime of the host (40, 41). Host factors such as nutrient metabolism and immune status can impact gut microbiome homeostasis significantly (42). During the aging process, biological and physiological functions systemically decline, leading to a loss of immune and metabolic homeostasis (3). Thus, we might predict that the gut microbiota would undergo both compositional and functional alterations. Considering the important roles described for the gut microbiome in numerous disease processes, many of which are related to aging (43, 44), determining how the composition and function of the gut microbiome change in response to aging, and how these may differ in individuals with healthy versus unhealthy aging, is of great interest. Robust characterization of age-related changes is critical for developing microbiome-based antiaging strategies (e.g., dietary intervention) and identifying therapeutic targets to promote health and longevity in the elderly.

In this meta-analysis, we aimed to define aging-related gut microbiome alterations shared across a number of independent mouse studies. Our study found that the aged murine gut microbiome is characterized by increased alpha diversities. Microbial diversity was reported to be decreased with aging in a few human studies (4, 5), but this decrease is not consistently observed. Odamaki et al. reported increased diversity in the elderly Japanese population compared to young adults (45), while no difference in alpha diversity was observed between young and elderly Italian populations (11). The variability across human studies might be due to the different patterns of aged gut microbiomes between healthy and relatively less healthy populations as well as between-population differences in external aging-associated factors impacting the gut microbiome. For example, diet, medication, disease status, and duration of stay in a care facility may all impact the aging microbiome, yet data on these factors are frequently lacking in human aging microbiome research (15, 46). Although decreased microbial diversity is associated with several diseases (47), higher diversity does not necessarily imply a healthier microbial community. Additional metrics to describe microbial stability, functional diversity, and components that benefit the host need to be taken into account in aging microbiome research. Thus, the biological meaning of the increased diversity in response to aging requires further investigation.

We further characterized differences in young and old microbial communities. Interestingly, we found a significant age-dependent shift of the microbial community based on unweighted UniFrac distance but not weighted UniFrac distance. As unweighted UniFrac distance takes only absence/presence information into account regardless of the relative abundance (48), the results suggest that rare taxa rather than dominant taxa drove the microbial structure differences between young and old mice. This is consistent with the finding of unaltered F/B ratios between young and aged mice. Instead, we found that a group of rare or subdominant taxa was differentially enriched (e.g., *Odoribacter* and *Mucispirillum*) or depleted (e.g., *Akkermansia*) in response to aging. It is interesting that these taxa are also commonly observed to be altered with aging in human studies (10, 11, 49, 50) and have been implied to be beneficial microbes or pathobionts. *Odoribacter* has been previously reported as an unfavorable species for metabolism, associated with abnormal lipid and glucose metabolism in the host (51, 52). Both *Mucispirillum* and *Akkermansia* degrade mucin, yet while *Akkermansia* is regarded as a beneficial microbe (53), the health relevance of *Mucispirillum* is still controversial (54). *Akkermansia* supports gut barrier integrity and contributes to anti-inflammation and antioxidation in aging (55, 56). *Mucispirillum* has been associated with intestinal inflammation in a few studies (57–59), while a protective role of *Mucispirillum* has been studied in Salmonella colitis in mice (60).

Alterations in rare taxa alone would not necessarily drive significant changes in microbial function. To examine whether aging alters the functional capacity of the gut microbiome, we next examined community-level microbial function using the bioinformatic tool PICRUSt2 to predict microbial metagenome function. We then established a random forest classifier to differentiate microbiomes from young and old animals and to identify aging-specific metabolic features. A random forest classifier is an ensemble of decision trees that uses random selection of different subsets of the features in the training data for constructing the different base classifiers (61). Unlike a single-decision tree, a random forest averages weak learners and avoids putting too much weight on outlier decisions (62). Random forest is thus one of the most robust classification algorithms developed to date and is used extensively in biomedical domains (63). However, when there is a large number of features and the proportion of truly informative features is small, the performance of a random forest classifier is likely to decline due to the low probabilities of choosing truly informative features by random sampling. Therefore, we applied an iterative random forest algorithm to construct the random forest classifier (64). The algorithm starts with the entire set of metabolic features in the training data set and stores the importance (mean decrease in Gini impurity) of the metabolic features. In the next iteration, a new weighted random forest is grown with weights set to the feature importance from the previous iteration. Ten iterations were performed in our study. Considering the limitations of using predicted metagenomic function rather than direct metagenome sequencing to construct the random forest classifiers, we included one 16S rRNA gene sequencing and one whole-genome sequencing data set generated by our laboratory as external data sets to validate the final random forest model. The performance of our random forest classifier in differentiating between microbiomes from young and old mice was good, with obtained AUC values of between 0.75 and 0.97 for the test data set and the two external data sets. Thus, the features selected by the random forest classifier appear to be informative and reliable aging-specific metabolic features.

Interestingly, we found that 50% of the 20 most important features identified by the random forest classifier are involved in carbohydrate metabolism. Bacterial carbohydrate metabolism is a basic biological process providing energy and precursors for biosynthetic pathways. Additionally, metabolites derived from microbial carbohydrate metabolism, primarily short-chain fatty acids (SCFA), exert biological effects on the host (38). The altered carbohydrate metabolism potential that we observed in aged mice potentially affects the production of SCFA. In fact, we found that fecal acetate, propionate, and butyrate were significantly decreased in the aged mice, which is consistent with data from other published mouse studies (26, 27).

GPR41 and GPR43 are the primary receptors that sense SCFA and mediate the biological effect of SCFA on host health (37). GPR41 prefers the longer SCFA (butyrate ≥ propionate > acetate), while GPR43 responds more strongly to the shorter SCFA (acetate ≥ propionate > butyrate) (37). The decreased *Gpr41* expression and increased *Gpr43* expression in aged mice suggest that host-microbiome communication could be altered with aging both by changes in the abundance of SCFA and by the decreased ability to sense longer SCFA (e.g., butyrate and propionate). The correlation of colonic *Gpr41* expression in the host with the abundance of gut microbes, microbial carbohydrate degradation and fermentation metagenomic potential, and fecal SCFA suggests that changes in the gut microbiome-SCFA with aging may in turn impact GPR41 and downstream host pathways. One potential downstream effect of altered SCFA-GPR41/43 signaling is inflammation, and thus, we examined two cytokines reported to be increased with aging. Despite a relatively small data set, we detected a trend toward increased *Il-6* gene expression in the colon as well as increased serum TNF-α in aged mice, suggesting an increased inflammatory state in aged mice. However, the extent to which changes in the gut microbiome-SCFA-GPR41 axis with aging impact host physiology and health remains to be elucidated.

There are several limitations of our study. To generate comparable data sets, a closed-reference OTU-picking process was chosen. This method restricts OTUs identified within the reference database (Greengenes V13-8), which excludes OTUs that are not present in the database and biases toward lower microbial diversity. Moreover, taxonomic assignments between studies may be influenced by the differences in sequencing lengths and regions between studies, which potentially introduce noise across the data sets (65). In addition to these technical issues, host-related covariates could also contribute to variations across the studies, which we were not able to control due to the missing individual-level metadata of aging-associated biological factors (e.g. metabolic and inflammation status [16, 66]). Furthermore, aging is a gradual process, and gut microbiome alteration with aging is likely to also be a gradual process in mice. However, due to the limited data available from published studies, we were able to include samples at only two ages bookending the spectrum of aging (young versus old). Additionally, we defined a relatively wide range for old (18 to 26 months of age). Thus, we lacked the ability to detect differences between moderately and extremely old mice and potentially introduced variability in our aged cohort that could decrease our ability to detect true differences between young and old mice. Finally, we cannot exclude the possibility that the observed correlation of host *Gpr41* expression with the gut microbiome is driven by age-related changes in both. Due to the limited age variation of our samples, we were unable to effectively control for age as a covariate in our Spearman correlation analysis. In the future, we hope to expand this work to include a variety of ages across the life span, which would facilitate investigations of whether correlations between changes in gut microbiome features and alterations in host physiology are independently correlated.

Despite these limitations, our study revealed both compositional and functional alterations in the gut microbiome with aging. Altered carbohydrate metabolism was a key feature of aging-related changes in the gut microbiome and was accompanied by decreased fecal SCFA. On the host side, colonic *Gpr41* expression was significantly correlated with microbial carbohydrate degradation and fermentation pathways. These findings show that compositional changes in the gut microbiome with aging correlate with functional changes, leading to measurable effects on key metabolites, as well as age-related changes in host receptors for those metabolites and imply cross talk between the gut microbiome, microbial SCFA, and host GPR41-dependent pathways in response to aging. We hope that the results from this meta-analysis across 5 independent studies will be a foundation for future work to further advance our understanding of the aging microbiome and host interaction from a putative correlation to a causative relationship and, ultimately, translation to clinical applications such as microbial manipulation to improve healthy aging and prevent age-related diseases.

## MATERIALS AND METHODS

### Data sets.

Literature searches of PubMed and Google Scholar were executed in July 2020 using the following keyword terms: “aging microbiome” OR “aging microbiota” OR “aged microbiome” OR “aged microbiota” OR “old microbiome” OR “old microbiota” AND “murine” OR “mice” OR “mouse.” The search yielded 35 potential studies for inclusion. We excluded studies where raw sequencing data were not publicly available, the sample number was <3 per age group, the average sequencing depth was <10,000 reads per sample, or the study groups did not meet our definition of young (1 to 6 months old) or old (18 to 26 months old). We also excluded studies of genetically modified or diseased animals (see Fig. S1 in the supplemental material).

10.1128/mSystems.01248-21.1 FIG S1 Data search and selection. Download FIG S1, TIF file, 1.6 MB. Copyright © 2022 You et al. 2022 You et al. https://creativecommons.org/licenses/by/4.0/ This content is distributed under the terms of the Creative Commons Attribution 4.0 International license.

After filtering, 5 studies that fulfilled our criteria were included in the meta-analysis. One study included data from both female and male C57BL/6 mice (18), and these were analyzed separately for a total of 6 data sets: 3 from female (16–18) and 3 from male (18–20) C57BL/6 mice. Within each data set, we further filtered out individual samples from animals that had a low sequencing depth (<2,000 reads) or were manipulated in any way (i.e., dietary intervention). The raw sequencing data were downloaded from the SRA and ENA databases by accession number, as listed in Table S1. As external data sets for validation of the random forest classifier, we included one fecal whole-genome metagenomics data set from male C57BL/6 mice sequenced as described below (NCBI SRA accession number PRJNA739153) and one 16S rRNA gene data set from male CB6F1 mice previously sequenced by our laboratory (You X, Yan J, Herzog J, Campbell R, Hoke A, Hammamieh R, Sartor RB, Kacena MA, Chakraborty N, Charles JF, manuscript in preparation) (NCBI SRA accession number PRJNA737742).

10.1128/mSystems.01248-21.7 TABLE S1 List of samples and associated accession numbers. Download Table S1, XLSX file, 0.02 MB. Copyright © 2022 You et al. 2022 You et al. https://creativecommons.org/licenses/by/4.0/ This content is distributed under the terms of the Creative Commons Attribution 4.0 International license.

Thus, in total, 3 female C57BL/6 data sets (*n* = 32) and 3 male C57BL/6 data sets (*n* = 102) were included in the meta-analysis and the generation of the random forest classifier. External data sets for validation of the random forest classifier included one male CB6F1 16S rRNA gene sequencing data set (*n* = 16) and 1 male C57BL/6 whole-genome metagenomics data set (*n* = 20).

### OTU picking.

Due to the variant amplicons of the 16S region that were sequenced across studies, we chose a closed-reference OTU-picking process to ensure concordance between studies. The raw sequencing data were imported into QIIME2/2018.11 for data processing (67). DADA2 was used to denoise reads (68). The reads were trimmed such that the mean quality score was <30 for four consecutive reads or the minimal sequencing length. For paired reads, reads were merged if the overlapped reads were >20 bp after truncation. Otherwise, only forward reads were used for the following analysis. Due to the potentially insufficient removal of the adapter/index/primer in the raw sequencing data, which would interfere with DADA2’s error model, we also trimmed 16 nucleotides from the 5′ end of each read. The quality-controlled, dereplicated reads were then subjected to OTU picking by q2-vsearch against the Greengenes V13-8 database at 97% identity.

### Diversity analysis.

Six OTU tables generated from the q2-vsearch clustering process were combined. OTUs with fewer than 5 total reads or that presented in only one sample were filtered out. Samples were rarefied to 10,000 reads by the rrarefy function in the R package Vegan (2.5-6) (69). Shannon, Simpson, and Chao1 indices were calculated by the diversity and estimateR functions in Vegan. Faith’s phylogenetic distance was calculated by the R package Picante (1.8.2) (70). Welch’s *t* test was performed per study analysis using the function t.test (stats, R package). The combined studies were analyzed using a linear mixed-effects model by the lmer function in the R package LmerTest (3.1-3) (71) using the formula log_2_FC ∼ age + (1|study). The same formula was also applied to the enrichment analysis of centered log-ratio normalization of OTUs and predicted PICRUSt2 metagenomic functional pathways (PICRUSt2 method [see below]). Weighted UniFrac and unweighted UniFrac distances were calculated by the R package Phyloseq (1.32.0) (72). PERMANOVA with 999 permutations (vegan::adonis) was performed for the differences in beta diversities. For combined studies, PERMANOVA was performed with the option strata to control for study.

### Fecal DNA extraction and whole-genome microbial sequencing.

Individual fecal samples were collected from 10 young (2-month-old) and 10 old (26-month-old) C57BL/6 male mice (NIA Aged Rodent Colony, USA), under IACUC approval 20110 (Indiana University). Genomic DNA was isolated from fecal samples using a QIAamp PowerFecal Pro DNA kit (Qiagen, MD, US) according to the manufacturer’s protocol. Fecal DNA was diluted to 10 ng/μL and submitted to the Broad Institute (MA, US) for high-output whole-genome microbial 150-bp paired-end sequencing. After sample DNA quality assessment, sequencing libraries were prepared using the Illumina Nextera XT DNA library prep kit. Two positive controls and one negative control were included in the sequencing run. In total, 1126.04 million reads (56.30 million ± 12.42 million reads per sample) were generated (Fig. S4).

10.1128/mSystems.01248-21.4 FIG S4 Whole-metagenome sequencing of young and old mice. (A) Sequencing reads across the samples. (B) Average sequencing depth in young and old mice after filtering out mouse genome contaminant reads. (C) Percentage of sequencing reads retained for the downstream analysis. (D and E) Relative abundances of taxa at the phylum level (D) and the genus level (E) of the whole-genome sequencing data from young and old mice. Download FIG S4, TIF file, 1.9 MB. Copyright © 2022 You et al. 2022 You et al. https://creativecommons.org/licenses/by/4.0/ This content is distributed under the terms of the Creative Commons Attribution 4.0 International license.

### Whole-genome OTU picking and metagenomic functional analysis.

Unmapped BAM files were received from the Broad Institute Genomics Center. Picard (2.6.0) was used to convert BAM files to fastq files. Sequencing quality was checked by FastQC (0.11.8). Raw reads were adapter trimmed by Trimmomatic (0.39) (73) and decontaminated of the host genome (mouse GRCm38.p6) mapped by Bowtie2 (2.4.2) (74) as part of the Kneadata (0.7.4) pipeline using the default parameters (https://github.com/biobakery/kneaddata). To consistently apply the analysis pipeline to 16S data sets, we used an OTU-picking process to generate the OTU table. Filtered reads were selected by alignment against the Greengenes V13-8 database via SortMeRNA (v.4.3.4) (75). OTU picking on the resulting reads was then performed by q2-vsearch against the Greengenes V13-8 database at 97% identity. For metagenomic functional analysis, filtered reads were processed by the HUMAnN3 (3.0.0.alpha.3) pipeline for the metagenomic functional analysis, including the following steps: (i) taxonomic profiling by MetaPhlAn3 (3.0.7) (76), (ii) indexing of the identified species pangenomes in the ChocoPhlAn database and alignment of reads to the reference pangenomes by Bowtie2, (iii) translated searching of unmapped reads against the UniRef90 protein reference database using DIAMOND (2.0.6) (77), and (iv) mapping of the resulting gene lists to the MetaCyc pathways. The normalized relative abundances of OTUs and MetaCyc pathways were then used as an external data set for the validation of random forest classifiers as described below. Moreover, the taxon table obtained by MetaPhlAn3, which has a better taxon resolution than OTU picking, was used to perform Spearman correlation analysis.

### Random forest classifier.

PICRUSt2 (Phylogenetic Investigation of Communities by Reconstruction of Unobserved States) (78) was used to predict metagenomic functions based on the six 16S rRNA gene data sets described above in “Data sets.” The data sets of MetaCyc pathways and OTUs were used as predictor variables for the random forest classifier. The data set was randomly divided into training (*n* = 89) and testing (*n* = 45) data sets. The iterative random forest (iRF) algorithm was performed using the R package iRF (64). Ten iterations were performed, of which the random forest with the smallest out-of-bag (OOB) error rate was chosen.

The receiver operating characteristic (ROC) curve and the area under the ROC curve (AUC) were calculated for the testing data set by the prediction and performance functions in the R package ROCR (79). We then applied the same functions to the two external data sets generated in our laboratory and as described above in “Data sets” to validate the performance of the random forest classifier.

### LC-MS measurement of fecal SCFA.

Fecal SCFA quantification was performed by liquid chromatography-mass spectrometry (LC-MS) at the Georgetown University Medical Center. A 20 mM standard solution of acetic acid (C_2_), propionic acid (C_3_), and butyric acid (C_4_) was prepared in water. For derivatization, 100 μL each of 4-acetoamido-7-mercapto-2,1,3-benzoxadiazole (AABD-SH) (20 mM), triphenylphosphine (TPP) (20 mM), and 2,2′-dipyridyl disulfide (DPDS) (20 mM) were added to 100 μL of C_2_, C_3_, and C_4_, separately. The reaction mixture was vortexed for 5 min at room temperature, followed by drying under nitrogen. The dry concentrate was dissolved in 100 μL of methanol and serially diluted to generate standard curves.

Fecal samples were homogenized in 400 μL of water for 2 min on ice and centrifuged, and the supernatant was collected. For derivatization purposes, 20 μL each of AABD-SH, TPP, and DPDS were added to the supernatant and vortexed at room temperature for 5 min. The reaction mixture was dried under a vacuum and reconstituted with 150 μL of methanol containing lactic acid-^13^C_3_ as an internal standard. The samples were centrifuged, and the supernatant was diluted 10-fold.

Three microliters of the prepared sample was injected onto a Kinetex 2.6-μm, 100-Å, 100- by 3.0-mm polar C_18_ column (Phenomenex, CA, USA) using a SIL-30 AC autosampler (Shimadzu) connected to a high-flow LC-30AD solvent delivery unit (Shimadzu) and a CBM-20A communication bus module (Shimadzu) online with a Qtrap 5500 instrument (Sciex, MA, USA) operating in positive-ion mode. A binary solvent comprising water with 0.1% formic acid (solvent A) and acetonitrile with 0.1% formic acid (solvent B) was used. The extracted metabolites were resolved at a 0.4-mL/min flow rate. MS was conducted in positive-ion mode with a turbo ion spray voltage of 5,500 V, using 20 lb/in^2^ of curtain gas, 50 lb/in^2^ of nebulizer gas, and 50 lb/in^2^ of drying gas at a temperature of 400°C. LC separation was performed using mobile phase A (0.1% formic acid in water) and mobile phase B (0.1% formic acid in acetonitrile) at a flow rate of 400 μL/min and a temperature of 40°C. The separation gradient was as follows: 0% B at 0 min, 0 to 100% B in 8 min, 100 to 0% B in 1 min, and 0% B in 1 min. A collision energy of 15 V was used for multiple-reaction monitoring (MRM), and LC-tandem MS (LC-MS/MS) data were analyzed by Analyst 1.5.2 software (AB Sciex). The peak area of the lactic acid-^13^C_3_ isotope-labeled internal standard was used to normalize SCFA and processed using MultiQuant 3.0.3 software. The column was conditioned using the pooled quality control (QC) samples initially and after every 6 sample injections to monitor shifts in signal intensities and retention times. A blank solvent was run before and after the pooled QC samples to minimize carryover effects. The concentration data were calculated from standard curves performed by QC-based locally estimated scatter plot smoothing (LOESS) signal correction (QC-RLSC) followed by normalization to the input fecal weight.

### Colon RNA extraction, cDNA synthesis, and qRT-PCR.

Colon tissues were snap-frozen in liquid nitrogen and stored at −80°C until analysis. Total RNA was isolated by tissue homogenization with a bullet blender bead beater (Navy Rino RNA lysis kit; Next Advance) followed by an RNeasy minikit (Qiagen) according to the manufacturer’s protocol. DNA contamination was removed by DNase I treatment (Thermo Scientific). cDNA synthesis was performed using a high-capacity cDNA reverse transcription kit (Applied Biosystems). Quantitative real-time PCR (qRT-PCR) was performed using Fast SYBR green master mix (Applied Biosystems). The primer sequences of the target genes are listed in Table S2. The reactions were performed on a StepOne Plus real-time PCR machine (Applied Biosystems, USA). Results were normalized to the housekeeping gene hypoxanthine phosphoribosyltransferase (HPRT) and calculated by the 2^−ΔΔ^*^CT^* method.

10.1128/mSystems.01248-21.8 TABLE S2 Primers for qRT-PCR. Download Table S2, XLSX file, 0.01 MB. Copyright © 2022 You et al. 2022 You et al. https://creativecommons.org/licenses/by/4.0/ This content is distributed under the terms of the Creative Commons Attribution 4.0 International license.

### Serum TNF-α and IL-6 analysis.

Blood samples were collected by cardiac puncture. Serum samples were separated by clotting the blood samples for 30 min, followed by spinning for 10 min at 12,000 × *g*. Serum TNF-α and IL-6 were measured using a murine TNF-α standard 2,2′-azinobis(3-ethylbenzthiazolinesulfonic acid) (ABTS) enzyme-linked immunosorbent assay (ELISA) development kit and a murine IL-6 standard ABTS ELISA development kit (Peprotech) according to the manufacturer’s protocol.

### Statistical analysis.

Unless otherwise specified, statistical analysis was performed with GraphPad Prism 8. Data were represented as means ± standard deviations (SD). An unpaired *t* test (parametric data) or a Mann-Whitney U test (nonparametric data) was performed for fecal SCFA, colon gene expression, and serum TNF-α and IL-6. Spearman rank correlation was performed between colon gene expression and gut microbial carbon degradation/fermentation pathways, between colon gene expression and fecal SCFA, and between taxa and fecal SCFA. The adjusted *P* value was determined by the Benjamini-Hochberg method using the function p.adjust in the base R package for *P* values obtained from the Spearman rank correlation and enrichment analyses. A *P* value of <0.05 was regarded as statistically significant.

### Code availability.

The code used for the analysis in the paper is available at GitHub (https://github.com/xyou103/murine-aging-microbiome-meta-analysis).

### Data availability.

Raw sequencing data for each study can be accessed as listed in Table S1. The sequencing data generated in our laboratory has been deposited in the NCBI SRA under accession numbers PRJNA737742 (16S rRNA gene) and PRJNA739153 (whole-genome metagenomics).
