# Supplementary figures and images for: Murine Gut Microbiome Meta-analysis Reveals Alterations in Carbohydrate Metabolism in Response to Aging
Source: mSystems. 2022 Apr 11;7(2):e01248-21. doi: 10.1128/msystems.01248-21 (PMC9040766; doi:10.1128/msystems.01248-21)

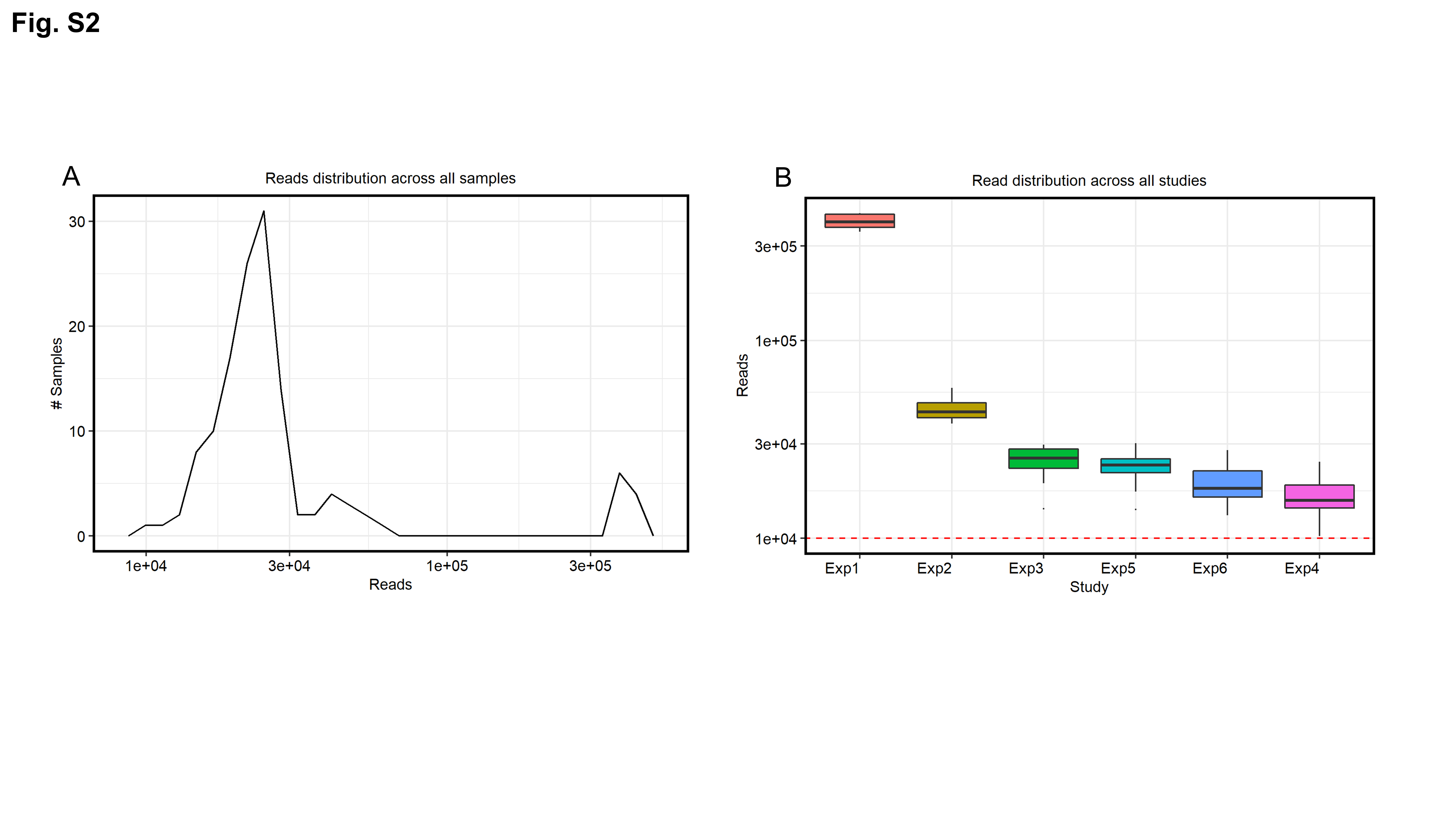

Supplement: FIG S2 [file msystems.01248-21-s0002.tif]

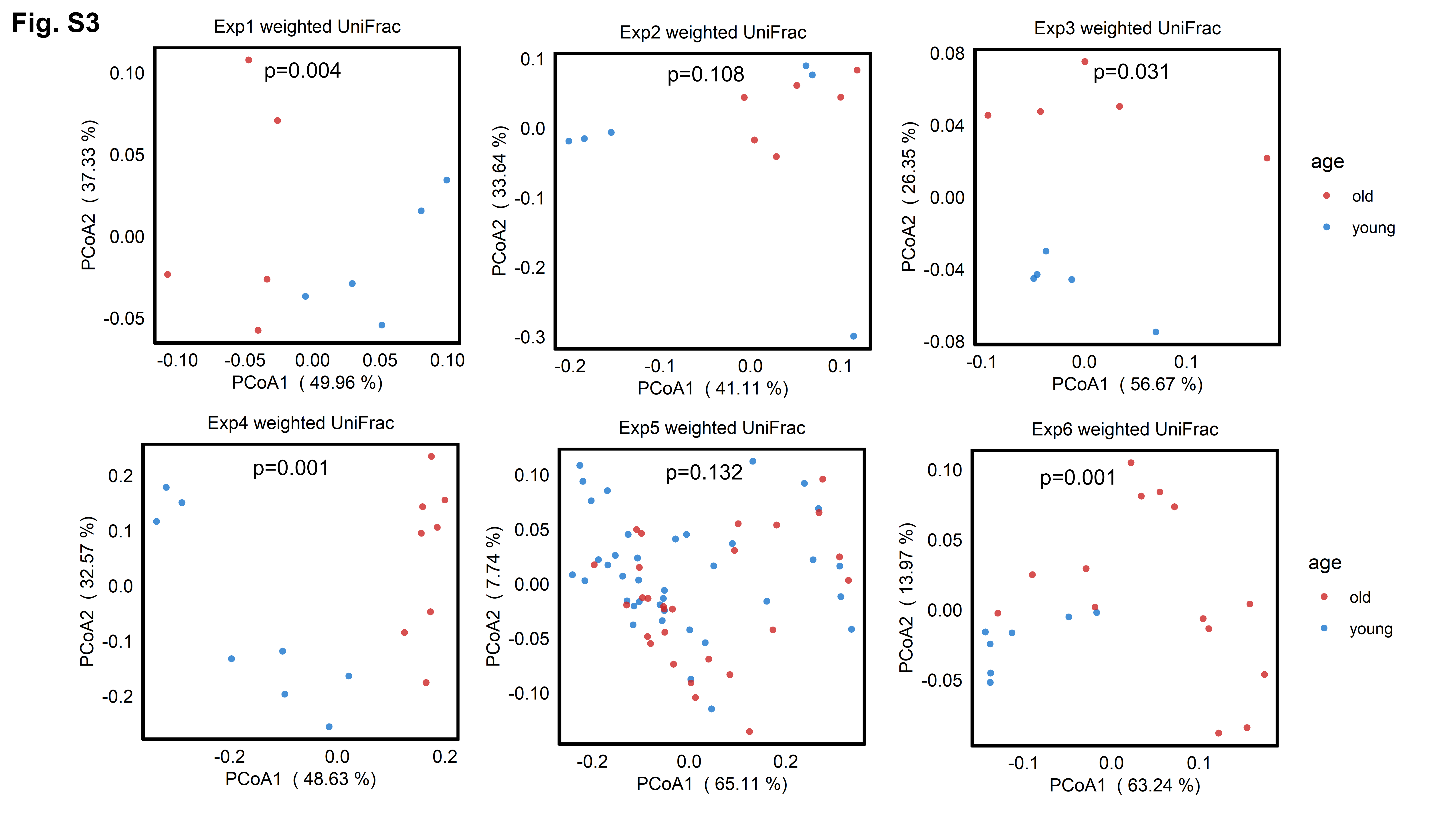

Supplement: FIG S3 [file msystems.01248-21-s0003.tif]

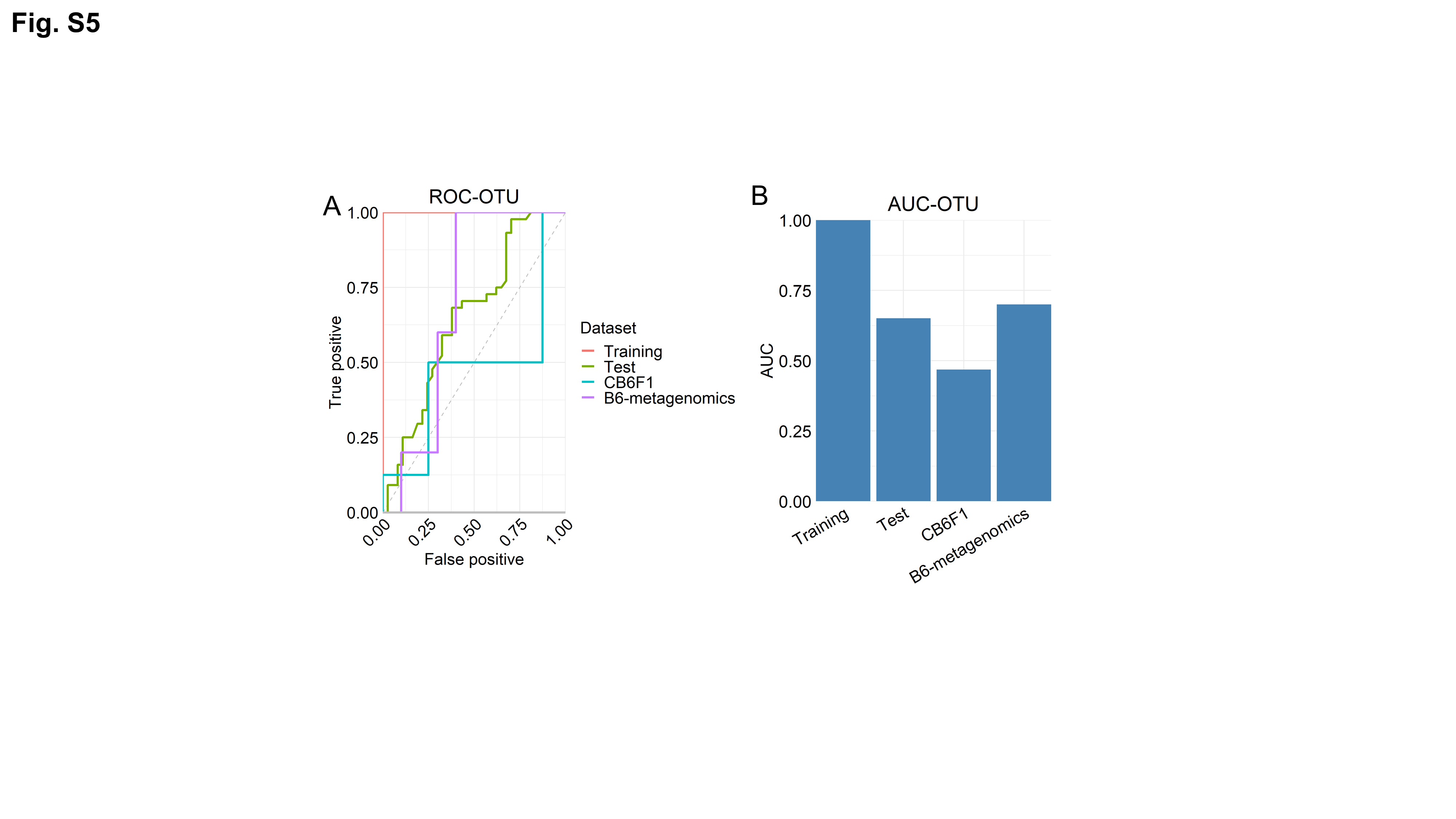

Supplement: FIG S5 [file msystems.01248-21-s0005.tif]

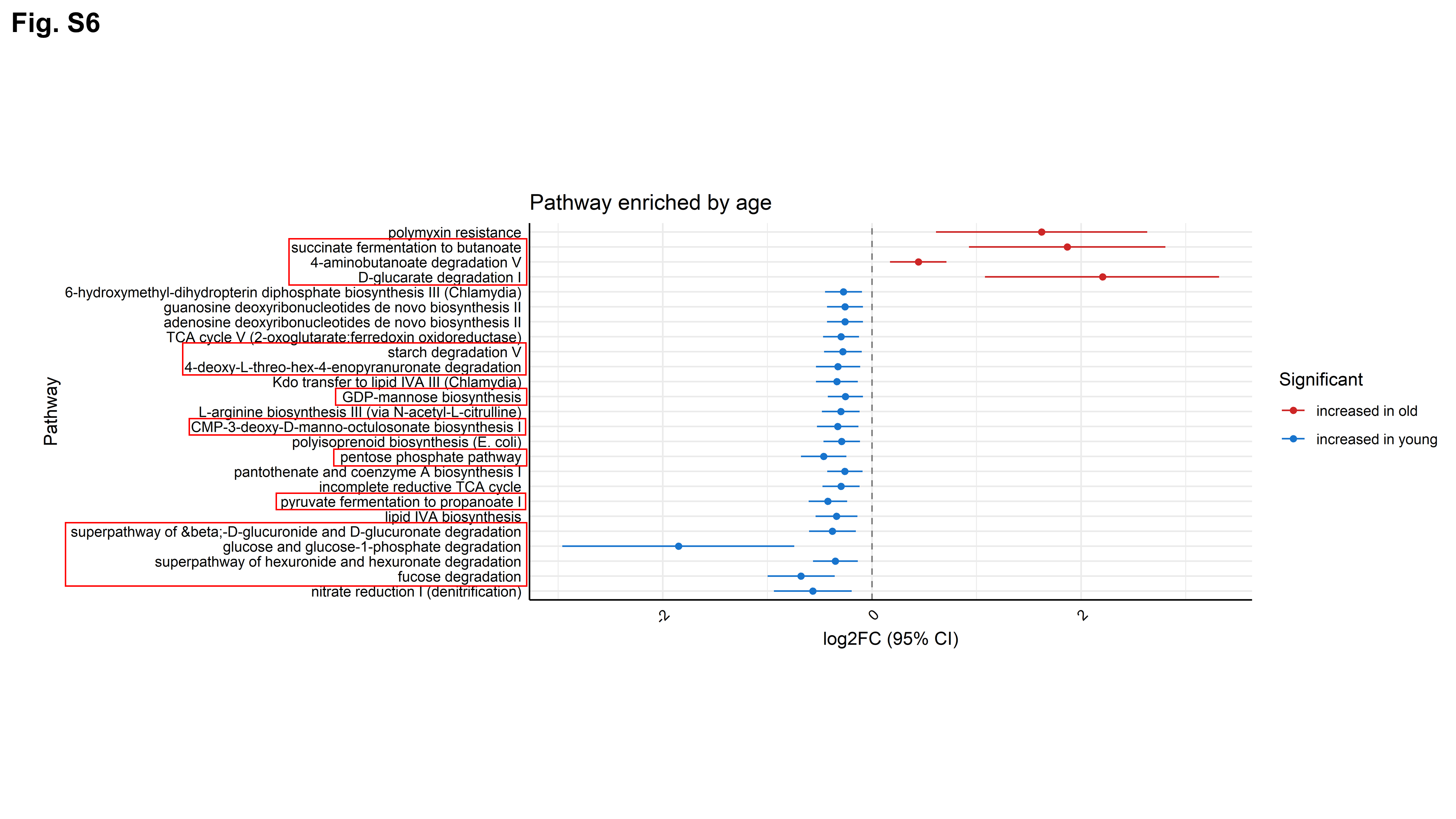

Supplement: FIG S6 [file msystems.01248-21-s0006.tif]

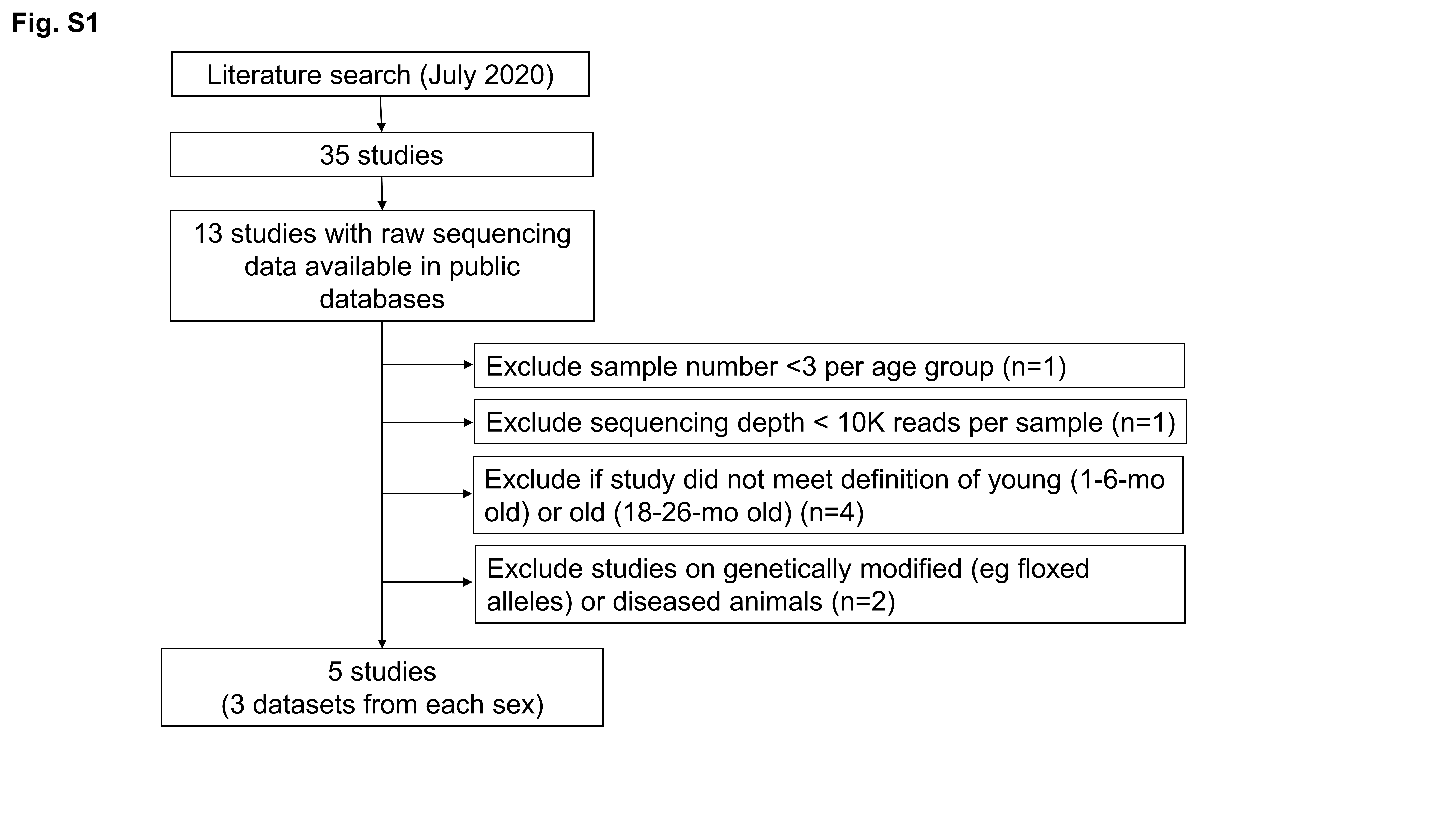

Supplement: FIG S1 [file msystems.01248-21-s0001.tif]

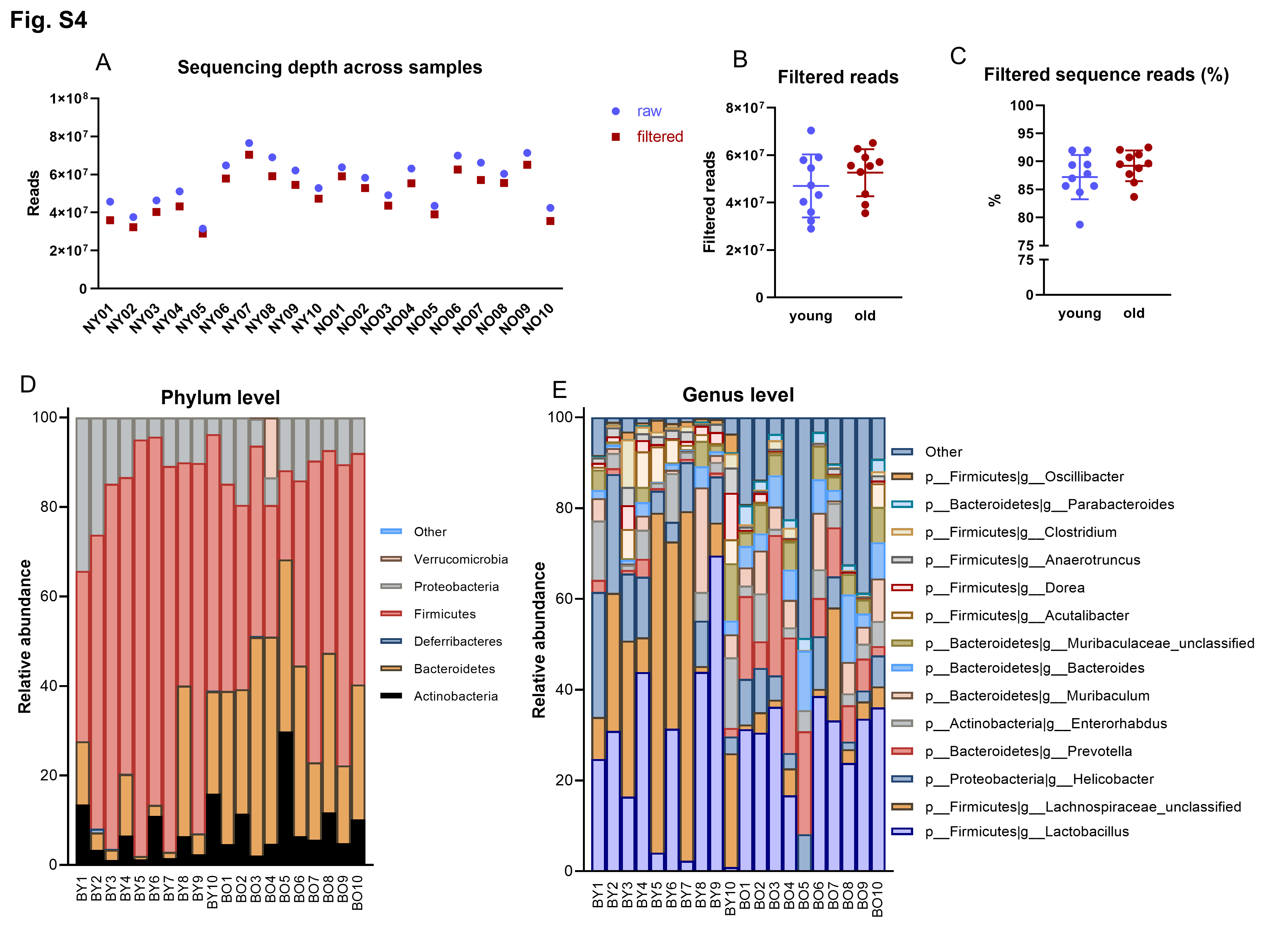

Supplement: FIG S4 [file msystems.01248-21-s0004.tif]
